# Draft Genome Sequence of *Weissella oryzae* SG25^T^, Isolated from Fermented Rice Grains

**DOI:** 10.1128/genomeA.00667-14

**Published:** 2014-07-10

**Authors:** Yasuhiro Tanizawa, Takatomo Fujisawa, Takako Mochizuki, Eli Kaminuma, Yutaka Suzuki, Yasukazu Nakamura, Masanori Tohno

**Affiliations:** a Department of Computational Biology, Graduate School of Frontier Sciences, the University of Tokyo, Chiba, Japan; b Genome Informatics Laboratory, National Institute of Genetics, Mishima, Japan; c Department of Medical Genome, Graduate School of Frontier Sciences, the University of Tokyo, Chiba, Japan; d National Agriculture and Food Research Organization, National Institute of Livestock and Grassland Science, Nasushiobara, Japan

## Abstract

*Weissella oryzae* was originally isolated from fermented rice grains. Here we report the draft genome sequence of the type strain of *W. oryzae*. This first report on the genomic sequence of this species may help identify the mechanisms underlying bacterial adaptation to the ecological niche of fermented rice grains.

## GENOME ANNOUNCEMENT

Rice production is the largest single use of agricultural land (over 11% of the total global cultivated area) and provides approximately 21% of global human per capita energy and 15% of per capita protein (1). Recently, there has been a growing interest in the special use of rice not only as food but also as a nutritional feed for livestock. *Weissella oryzae* SG25^T^ was originally isolated from fermented rice grains for livestock (2), suggesting that it is associated with adequate fermentation of rice grain silage and has the potential for biotechnological applications such as an effective inoculant for rice grains.

The genomic DNA of the strain SG25^T^ was extracted and purified from cells harvested from de Man, Rogosa, and Sharpe broth (Difco) using Qiagen Genomic-tips. Roche 454 pyrosequencing and Illumina HiSeq 2000 produced 169,062 single-end reads and 557,004,694 pair-end reads, respectively. The former reads were assembled into 109 contigs with Newbler assembler (version 2.7). The latter reads were first extended by an overlap of pair-end reads using the Flash program (3), followed by filtering for sequence quality and read length. Subsequently, the reads were aligned by Burrows-Wheeler Aligner (4) to the contigs obtained from Newbler, and the aligned sequences were subjected to the Columbus module of Velvet (version 1.2.08) (5) to perform alignment-assisted assembly. The obtained sequences were further improved by scaffolding using Opera (6) and by gap-closing using GapFiller (7). The closed gaps were manually verified. The final draft genome sequence consisted of 70 scaffolds. The genome was annotated using the Microbial Genome Annotation Pipeline online server (8), by which coding sequences and rRNAs and tRNAs were predicted, and database searches against RefSeq, TrEMBL, and clusters of orthologous groups (COGs) were conducted. The annotated genome was submitted to the GenomeRefine web service (http://genome.annotation.jp/genomerefine/), which refines annotation and assists registration to the International Nucleotide Sequence Database Collaboration through the DNA Data Bank of Japan.

The total length of the draft genome is 2,129,476 bp, with a G+C content of 39%. It contains 2,228 protein-coding genes, among which 1,264 have been assigned to specific COGs. It also contains 72 tRNAs, 5 copies of 5S rRNA, 1 copy of 16S rRNA, and 1 copy of 23S rRNA. This strain possesses gene clusters for the arginine deiminase (ADI) and agmatine deiminase (AgDI) pathways, both of which are believed to serve as alternative pathways to gain ATP and/or serve for acid tolerance by increasing cytoplasmic pH through NH_3_ production (9). Comparative analysis with other lactic acid bacteria revealed that the ADI pathway was similar to those found in the genus *Lactococcus* rather than those in the genus *Weissella* and the AgDI pathway was not found in other members of the genus *Weissella*, suggesting that they were acquired through horizontal gene transfer. These pathways may serve for a yet unidentified fermentation process of rice grains.

### Nucleotide sequence accession numbers.

This whole-genome shotgun project has been deposited at DDBJ/ENA/GenBank under the accession no. BAWR00000000. The version described here is the first version, BAWR01000000.
